# Mediating Effects of Emotional Self-Disclosure on the Relationship between Depression and Quality of Life for Women Undergoing In-Vitro Fertilization

**DOI:** 10.3390/ijerph18126247

**Published:** 2021-06-09

**Authors:** Miok Kim, Ju-Eun Hong, Minkyung Ban

**Affiliations:** 1 College of Nursing, Dankook University, Cheonan 31116, Korea; aprilsea@dankook.ac.kr; 2 Seoul Healing Center, Shym Pyo, Seoul City Hall, Seoul 04524, Korea; nurse0413@hanmail.net; 3 College of Nursing, Dong-A University, Busan 49201, Korea

**Keywords:** infertility, depression, self-disclosures, life quality

## Abstract

This study aimed to identify the moderating and mediating effect of emotional self-disclosure between depression and quality of life for women under infertility treatment. The subjects included 169 infertile women under in vitro fertilization (IVF) treatment. The data were collected by self-administered questionnaires from June to August in 2019. The questionnaire consisted of questions about depression, emotional self-disclosure, and fertility quality of life. Descriptive statistics, *t*-tests, one-way analysis of variance, correlation, and stepwise multiple regression were analyzed using the SPSS 25.0 Windows program. Depression had a negative correlation with emotional self-disclosure (r = −0.189, *p* = 0.014) and fertility quality of life (r = −0.532, *p* < 0.001). Emotional self-disclosure had a positive correlation with fertility quality of life (r = 0.259, *p* = 0.001). These results confirm that emotional self-disclosure has mediating effects between depression and fertility quality of life. Therefore, nursing interventions for IVF patients need to encourage expressing and sharing various emotions experienced through the diagnosis and treatment of infertility in order to alleviate negative emotions.

## 1. Introduction

Approximately 20% of married couples worldwide face the problem of infertility, which affects their daily living and impairs their quality of life (QOL) [1]. As a result, many studies have examined the QOL of women undergoing fertility treatment.

The success rate of assisted reproductive technology (ART, also as known as IVF) is below 30% [2], and women undergoing treatment not only suffer from physical difficulties, such as pain and discomfort, but may also experience various psychological and emotional challenges. With a weakened body and mind, women become highly vulnerable to the verbal influence of their family and health care providers and may experience negative interpersonal relationships. Such adverse experiences influence their future pregnancy motivation, willingness to engage in health self-care, thoughts about children, and work life [3]. According to reports, women who have undergone fertility treatment find mental pain, isolation, and family and social prejudice more agonizing than financial burdens [4].

Depression is a critical factor that erodes the QOL of women undergoing fertility treatment [5]. More than half of these women suffer from depression [6], and severe depression may diminish the success rate of ART [7] and impair their QOL [8]. It has been reported that the level of negative emotions is higher before initiating ART than during the in vitro fertilization (IVF) process [9], and the severity of depression is higher among marital couples undergoing repeated cycles than among those who are beginning their first cycle [10]. Furthermore, the risk for depression is high up to one year postpartum, even after successful conception via IVF and subsequent childbirth [11]. Women who have undergone IVF are at an elevated risk of depression 20–23 years later, regardless of their reproductive history [12], highlighting the need for robust emotional management for women preparing to undergo IVF.

As shown here, women receiving fertility treatment need social support as they experience negative emotions and impaired QOL. They dread the disclosure of their infertility in fear of criticism or potentially violating social norms [6]. In particular, Korean women, who have been coerced to suppress their emotional expression under Confucian values and a patriarchal society [13], experience a greater challenge in disclosing their infertility amidst South Korea’s sociocultural context. Thus, emotional self-disclosure [14], which facilitates post-traumatic growth by promoting desensitization via repeated disclosures of one’s emotions to alleviate the intensity of negative emotions linked to an event, should be utilized to mitigate the negative emotions experienced by women during the process of diagnosis and treatment of infertility, assisting them to achieve positive adjustment. In fact, the level of self-disclosure of infertility is associated with the degree of negative emotions [15]; hence, emotional self-disclosure appears to be important to attenuate negative emotional experiences.

For women who plan to undergo, or are currently undergoing, procedures to conceive, depression may have a direct and indirect impact, not only on their physical health, but also on their QOL and pregnancy-related outcomes. It may further influence their spouse and family, thereby triggering conflict. In this context, the use of a positive self-disclosure mechanism is anticipated to promote self-understanding, reduce depression, and improve QOL. This study aimed to investigate the moderating and mediating effects of emotional self-disclosure on the relationship between depression and QOL in women undergoing fertility treatment. The outcomes of this investigation may ultimately assist women to accept their situations more positively, and we present foundational data for developing nursing interventions to improve the QOL of women undergoing fertility treatment.

The objectives of this study are to assess the level of depression, quality of life, and emotional self-disclosure and to identify the moderating and mediating effects of emotional self-disclosure between depression and fertility quality of life for women undergoing infertility treatment.

## 2. Materials and Methods

### 2.1. Study Participants

Women undergoing IVF at one of two infertility clinics in Seoul and Cheonan, were selected for participation using a convenience sampling strategy. The sample size was determined using the G*power 3.10 program for the statistical tests used in this study. For regression analysis, the minimum sample size was calculated to be 139 for a significance level (*α*) of 0.05, power (1-*β*) 80%, medium effect size (*f*^2^) 0.15, and 15 predictors. Thus, 180 questionnaires were distributed in consideration of potential withdrawals. After excluding those who withdrew from the study, and those who made mistakes on the questionnaires, a total of 169 participants were included in the analysis. Thus, the sample size requirement was met. The general characteristics of participants are described in Table 1.

### 2.2. Data Collection

After obtaining approval from the institutional review board (approval number NSU-201712-009), data were collected from June to August 2019, from two infertility clinics in Seoul and Cheonan. The researchers informed the participants of the purpose and method of the study, compensation, anonymity, and confidentiality; only those who signed a written consent form were enrolled. Data were collected using a self-report questionnaire consisting of approved instruments, and the completed questionnaires were collected by the researcher and stored. The questionnaire required 10 min for completion, and the study participants were compensated with a small gift.

### 2.3. Study Instruments

#### 2.3.1. Depression

Depression was measured using the 20-item Korean version of the Center for Epidemiologic Studies Depression Scale (CES-D) as adapted, standardized, and validated by Cho and Kim [16], based on the original CES-D developed by the National Institute of Mental Health in 1971 to survey depression in the community. This tool measures the severity of depressive symptoms in the past week on a 4-point scale: 0 (extremely rare; ≤ 1 day in the past week), 1 (occasionally; 1–2 days in the past week), 2 (frequently; 3–4 days in the past week), and 3 (most of the time; ≥5 days in the past week). The total score ranges from 0 to 60, with a higher score indicating a higher level of depression. The most widely used cutoff points for the CES-D are 16 and 25, where a score of ≥16 indicates probable depression and a score of ≥25 indicates definite depression. The reliability of the tool (Cronbach’s α) was 0.90 in the general population (*n* = 540), 0.93 in the clinical patient group (*n* = 164), and 0.89 in the major depression group (*n* = 46) in the study by Cho and Kim [16]. The Cronbach’s α was 0.95 in this study on women undergoing IVF.

#### 2.3.2. QOL

QOL was measured using fertility quality of life (FertiQol) International, a questionnaire about the QOL of people with infertility proposed by the European Society of Human Reproduction and Embryology and the American Society of Reproductive Medicine [17]. It comprises 24 items covering infertility problems (6 items for emotion, 6 items for body and mind, 6 items for relationship, 6 items for social aspect), and 2 items about general QOL. Each item is rated on a 5-point scale, and a higher score indicates a higher QOL. The reliability of the tool (Cronbach’s α) was 0.92 (0.75–0.90) at the time of development, and 0.92 in this study.

#### 2.3.3. Emotional Self-Disclosure

Emotional self-disclosure was measured using the Distress Disclosure Index (DDI), originally developed by Kahn and Hessling [18], and adapted by Song and Lee [19]. The tool consists of 12 items that measure whether an individual expresses their negative emotions toward other people, and whether they tend to disclose or conceal emotions and thoughts provoked by a painful event. Some examples of the items include, “When I feel upset, I usually confide in my friends” and “When I am in a bad mood, I talk about it with my friends.” The Cronbach’s α was 0.93 in the study by Song and Lee [19] and 0.89 in this study.

### 2.4. Data Analysis

The collected data were analyzed using SPSS WIN 26.0 software (IBM Corp., Armax, NY, USA). Participants’ general characteristics, level of depression, emotional self-disclosure, and QOL were analyzed using descriptive statistics, and the relationships among the major variables were analyzed using Pearson correlation coefficients. The moderating and mediating effects of emotional self-disclosure on the relationship between depression and QOL in women undergoing fertility treatment were analyzed using hierarchical regression.

## 3. Results

### 3.1. Level of Depression, Emotional Self-Exposure, and QOL

The mean depression score was 1.28 (±0.42) on a scale of 0–3. Based on the CES-D cutoff, 0 (0.0%) were within the normal range (0–15), 112 (66.3%) had probable depression (≥16), and 57 (33.7%) had definite depression (≥25). The mean emotional self-disclosure score was 3.15 (±0.83) on a scale of 1–6. QOL was 3.42 (±0.61) on a scale of 1–5 (Table 2).

### 3.2. Correlations among Depression, Emotional Self-Exposure, and QOL

There was a negative correlation between depression and emotional self-exposure (r = −0.189, *p* = 0.014) and between depression and QOL (r = −0.532, *p* < 0.001), and a positive correlation between emotional self-exposure and QOL (r = 0.259, *p* = 0.001) in women undergoing IVF (Table 3).

### 3.3. Moderating and Mediating Effects of Emotional Self-Disclosure in the Relationship between Depression and QOL

In the moderation model, a factor Z is deemed a moderator if it affects the association between X and Y only under certain conditions of X and Z [20]. Under the assumption that emotional self-disclosure interacts with depression and improves QOL, people with low emotional self-disclosure have poor QOL, while those with high emotional self-disclosure have high QOL.

In the hierarchical regression analysis, R^2^ increased from 27.9% in Model 1, to 30.1% in Model 2, and 30.8% in Model 3, yet the F statistic was greater than 0.05, at 0.1; hence, the moderating effect could not be determined (Table 4). Therefore, we can conclude that the level of emotional self-disclosure had no influence on the relation between depression and QOL.

In the mediation model, a mediator Z is placed in the causative path between the predictor X and dependent variable Y, and the fact that a mediator is present means the predictor increases the mediator, which in turn affects the dependent variable in a causative path [20].

The mediating effects of emotional self-exposure on the relationship between depression and QOL were analyzed using three-step regression, as proposed by Baron and Kenny [20] (Table 5). In the first regression analysis to analyze the effects of depression on emotional self-exposure, *β* was −0.189 and statistically significant (t = −2.491, *p* = 0.014). In the second regression analysis to analyze the effects of depression on QOL, *β* was −0.532 and statistically significant (t = −8.120, *p* < 0.001). In the final regression analysis to analyze the effects of emotional self-exposure on QOL, *β* was 0.164 and statistically significant after adjusting for depression (t = 2496, *p* = 0.014). In the third step, the *β* value of depression was −0.501, which was smaller than that in the second step (−0.532), confirming that emotional self-exposure mediated the relationship between depression and QOL (t = −7.625, *p* < 0.001). The percentage of variance explained was 3.0% in step 1, 27.9% in step 2, and 30.1% in step 3. Therefore, we can conclude that emotional self-disclosure had a partially mediating effect between depression and QOL; depression affected emotional self-disclosure, and emotional self-disclosure, in turn, affected the QOL.

As described above, we find that self-disclosure played a partially mediating role but not a moderating role. This finding reveals one way by which depression influences QOL: through a lack of emotional self-disclosure. However, we also found that self-disclosure was only a partial mediator, suggesting that there are other, perhaps more direct paths, from depression to QOL.

## 4. Discussion

In this study, the degree of emotional self-disclosure was negatively correlated with depression and positively correlated with QOL, suggesting that it can help women undergoing fertility treatment to cope with, and better adjust to, their situations by relieving their anxiety and tension [19], ultimately having a positive impact on their QOL. Further, emotional self-disclosure was found to mediate the relationship between depression and QOL in women undergoing fertility treatment, confirming that emotional self-disclosure can help to counterbalance the negative effect of depression on QOL.

Infertile individuals have difficulties in disclosing their infertility problems, as they experience sociocultural pressure and fear social prejudice and negative views [21]. In particular, a high level of stigma experienced by women undergoing IVF is associated with poor QOL [22]. Due to the stigmatizing nature of infertility, women conceal their negative emotions because of the diagnosis itself, and during the treatment process, perceiving it as something to be endured alone, which isolates them from their families or social relationships [21]. In this context, emotional disclosure helps reduce mental health problems [19] and will counterbalance the adverse impact on QOL.

In this study, the mean depression score was 1.28 on a scale of 0–3 in women undergoing IVF, and approximately 64.5% of the women were deemed to suffer from probable depression or definite depression according to the CES-D criteria. This score (1.63) was lower than that obtained using the same instrument by Kim, Hong, and Lee [23] in women undergoing infertility treatment, where all participants of the previous study [23] experienced probable or definite depression. Infertility treatment takes the following three stages: ovulation induction, intrauterine insemination, and IVF. Ovulation induction involves taking oral or injectable medication to stimulate regular ovulation. In intrauterine insemination, the sperm is introduced into the uterine cavity. The last stage is IVF (assisted reproductive technology), where sperm and eggs are combined outside the woman’s body. Typically, each stage is undertaken when the previous stage has no positive results.

As 59.6% of women undergoing IVF and 57.0% of women undergoing intrauterine insemination are reported to experience mental pain, isolation, and depression [24], the frequency of negative emotions or level of depression does not differ according to the stage of infertility treatment [23]. However, as shown by the higher level of negative emotions before beginning IVF compared to that during the IVF process [9], the degree of negative emotions may differ during the procedures, calling for more sophisticated study designs that account for the stage of infertility treatment, such as intrauterine insemination and IVF, and the process of the procedures. Depression is a critical factor that may hinder infertility treatment, reduce the probability of successful conception, and result in the termination of treatment in women undergoing infertility treatment [25]; therefore, a systematic review of the predictors of depression and aggressive intervention strategies that alleviate depression are required.

The infertility-related QOL in the participants was 3.42, which was at a similar level (3.82) reported by Kim, Park, and Nam [26] using the same instrument. This is contextually in line with the negative correlation between depression and QOL and is consistent with a previous report that depression has a negative impact on QOL [27]. The diagnosis of infertility itself and the process of treatment is a long-term endeavor that provokes stress, which in turn induces depression and degrades QOL [28]. QOL in women undergoing IVF tends to be lower among those with a high level of depression, a history of treatment failure, and infertility of unknown cause. The cause of infertility and a history of treatment failure are important factors contributing to impaired QOL [27]. Thus, differentiated intervention strategies are required to adapt to different causes of infertility, and additional psychological and emotional interventions may be required for women who have undergone unsuccessful fertility treatments.

The mean emotional self-disclosure score in our participants was 3.15 on a scale of 1–6. While it is difficult to compare our results with those of the same target group due to the lack of studies that investigated emotional self-disclosure in women undergoing fertility treatment, this score (3.13) is similar to that reported among female family caregivers of adult cancer patients in the study by Kim [29]. Women experience trauma due to the diagnosis of infertility itself and due to the experience of miscarriage during the process of fertility treatment [30]. Such psychological discomfort and difficulties may be mitigated by helping women understand their own emotions and appropriately expressing them verbally and behaviorally [23]. Emotional self-disclosure [19]—the act of expressing one’s own emotions through writing or disclosing them to others—diminishes negative emotions incurred by a stressful event, improves one’s subjective well-being, and fosters positive perceptions of health [31]. Stated differently, disclosing (as opposed to suppressing) emotions experienced during the entire process of fertility treatment will enable women to recognize their emotions, positively understand themselves, face the situation, and solve the problems at hand.

Prior to disclosing their infertility status, women undergoing IVF consider their conditions, and need for support from others, based on factors such as the nature of their social group, potential stigmatization, and the advantages and disadvantages of concealing or disclosing the information [32]. According to a study on women undergoing fertility treatment [33], indirect exposure through the media and other channels is negatively correlated with infertility-related QOL. Conversely, direct or gradual disclosure in person, that is verbally expressed and provides an opportunity to provide immediate responses, is positively correlated with overall QOL. Therefore, fertility specialists need to take note of this during interviews, when treating women receiving infertility treatments, and when developing and implementing emotional self-disclosure promoting programs. Particularly, these women typically try to protect themselves while waiting for the results after embryo transfer by distancing themselves from others and avoiding discussing pregnancy tests with them [32]; therefore, more active monitoring and support interventions are required during this period.

The findings of this study must be generalized with caution, as the participants were convenience-sampled from patients visiting one of two facilities for IVF, and the use of a self-report questionnaire leaves the possibility of misleading responses to sensitive questions. However, this study is significant in that it highlighted the importance of emotional self-disclosure in nursing interventions for women undergoing fertility treatment, by verifying the mediating effects of emotional self-disclosure in the relationship between depression and QOL in these women.

## 5. Conclusions

This study attempted to examine the moderating and mediating effects of emotional self-disclosure on the relationship between depression and QOL in women undergoing IVF. The results showed that emotional self-disclosure mediates between depression and QOL in these women. Women receiving fertility treatment find it difficult to disclose their infertility in South Korea’s Confucian culture; thus, they do not receive adequate social support to alleviate their negative emotions. Further, as they undergo repeated cycles of treatment, the severity of their psychological and emotional difficulties intensifies, which erodes their QOL. Emotional self-disclosure may improve these women’s QOL by counterbalancing the link between negative emotions and impaired QOL. Hence, nursing interventions for women receiving fertility treatment, especially women undergoing IVF, are strongly recommended to include programs that provide them with an opportunity to express various emotions experienced following the diagnosis and during the treatment of infertility.

## Figures and Tables

**Table 1:** General characteristics of subjects (*n* = 169).

Characteristics	Categories	*n*	(%)	Mean ± SD
Age (years)	<35	78	(46.2)	35.05 ± 4.51
	≥35	91	(53.8)	
Duration of marriage (years)	≤1	9	(5.3)	
	>1~≤3	57	(33.7)	
	>3~≤5	52	(30.8)	
	>5	51	(30.2)	
Employment	Yes	89	(52.7)	
	No	80	(47.3)	
Religion	Have	62	(36.7)	
	Don’t have	107	(63.3)	
Child	Have	22	(13.0)	
	Don’t have	147	(87.0)	
Factor of infertility	Unexplained	104	(61.5)	
	Female factor	46	(27.2)	
	Male factor	3	(1.8)	
	Complex factor	16	(9.5)	
Abortion experience after infertility treatment	Yes	38	(22.5)	
	No	131	(77.5)	
Treatment cost affordability	Hardly affordable	115	(68.0)	
	Medium affordable	42	(24.9)	
	Easily affordable	12	(7.1)	
Government subsidy for infertility treatment	Yes	93	(55.0)	
	No	76	(45.0)	
Level of spouse support	Active	111	(65.7)	
	Neutral	51	(30.2)	
	Passive	7	(4.1)	

**Table 2:** Depression, emotional self-disclosure, and QOL of subjects (*n* = 169).

Variables	Item Score	Range of Scale	Total Range	*n* (%)
	(Mean ± SD)			
Depression	1.28 ± 0.42	0~3	0~60	
Normal			0–15	0 (0.0)
Probable depression			16–24	112 (66.3)
Definite depression			25–60	57 (33.7)
Emotional self-disclosure	3.15 ± 0.83	1~6	12~72	
QOL	3.42 ± 0.61	1~5	26~130	

**Table 3:** Correlation between depression, emotional self-disclosure, and QOL (*n* = 169).

Variables	Depression	Emotional Self-Disclosure	QOL
	r (*p*)	r (*p*)	r (*p*)
Depression	1	−0.189 (0.014)	−0.532 (<0.001)
Emotional self-disclosure	-	1	0.259 (0.001)
QOL	-	-	1

**Table 4:** Moderating effect of emotional self-disclosure in the relationship between depression and QOL (*n* = 169).

Model	R	R^2^	Adj.R^2^	Relative Standard Error	Statistics Change				
					R^2^ Change	F-Change	Degree of Freedom 1	Degree of Freedom 2	Significant F Change
1	0.532 ^a^	0.283	0.279	0.53	0.283	65.936	1	167	<0.001
2	0.556 ^b^	0.309	0.301	0.52	0.026	6.228	1	166	0.014
3	0.566 ^c^	0.320	0.308	0.51	0.011	2.733	1	165	0.100

Model 1—^a^ Fitted value: (constant), Depression. Model 2—^b^ Fitted value: (constant), Depression, Emotional self-disclosure. Model 3—^c^ Fitted value: (constant), Depression, Emotional self-disclosure, Interaction.

**Table 5:** Mediating effect of emotional self-disclosure in the relationship between depression and QOL (*n* = 169).

Model	Dependent Factor	Independence (Mediating) Factor	B	S.E.	*β*	t (*p*)
1	Emotional self-disclosure	(Constant)	3.634	0.201		18.079 (<0.001)
		Depression	−0.019	0.007	−0.189	−2.491 (0.014)
	F = 6.206 (0.014), adj.R^2^ = 0.030, VIF = 1.000, Durbin-Watson = 2.031					
2	QOL	(Constant)	4.424	0.129		34.311 (<0.001)
		Depression	−0.039	0.005	−0.532	−8.120 (<0.001)
	F = 65.936 (<0.001), adj.R^2^ = 0.279, VIF = 1.000, Durbin-Watson = 2.059					
3	QOL	(Constant)	3.980	0.218		18.232 (<0.001)
		Depression	−0.037	0.005	−0.501	−7.625 (<0.001)
		Emotional self-disclosure	0.122	0.049	0.164	2.496 (0.014)
	F = 37.114 (<0.001), adj.R^2^ = 0.301, VIF = 1.037, Durbin-Watson = 2.027					

## References

[1] Baldur-Felskov B., Kjaer S.K., Albieri V., Steding-Jessen M., Kjaer T., Johansen C., Dalton S.O., Jensen A. (2012). Psychiatric disorders in women with fertility problems: Results from a large Danish register-based cohort study. Hum. Reprod..

[2] Hwang N.M., Lee S.H., Park S.M., Jang I.S., Kang A.L. (2016). Evaluation of supporting program for infertile couples in 2015 and causal analysis of infertility. Minist. Health Welf. Health Soc. Wlef..

[3] Hong J.-E., Park J.-M. (2017). A Phenomenological Study on the Spontaneous Abortion Experiences of Women. Korean J. Women Health Nurs..

[4] Statistics Korea (2015). Pregnancy and Childbirth Behavior of Married Women: 2015–2015.

[5] Shahraki Z., Tanha F.D., Ghajarzadeh M. (2018). Depression, sexual dysfunction and sexual quality of life in women with infertility. BMC Women’s Health.

[6] Namdar A., NaghiZadeh M.M., Zamani M., Yaghmaei F., Sameni M.H. (2017). Quality of life and general health of infertile women. Health Qual. Life Outcomes.

[7] Gdańska P., Drozdowicz-Jastrzębska E., Grzechocińska B., Radziwon-Zaleska M., Węgrzyn P., Wielgoś M. (2017). Anxiety and depression in women undergoing infertility treatment. Ginekol. Polska.

[8] Dural O., Yasa C., Keyif B., Celiksoy H., Demiral I., Ozgor B.Y., Ugurlucan F.G., Bastu E. (2016). Effect of infertility on quality of life of women: A validation study of the Turkish FertiQoL. Hum. Fertil..

[9] Massarotti C., Gentile G., Ferreccio C., Scaruffi P., Remorgida V., Anserini P. (2019). Impact of infertility and infertility treatments on quality of life and levels of anxiety and depression in women undergoing in vitro fertilization. Gynecol. Endocrinol..

[10] Reis S., Xavier M.R., Coelho R., Montenegro N. (2013). Psychological impact of single and multiple courses of assisted reproductive treatments in couples: A comparative study. Eur. J. Obstet. Gynecol. Reprod. Biol..

[11] Sejbaek C.S., Pinborg A., Hageman I., Forman J.L., Hougaard C. (2015). Ørsted; Schmidt, L. Are repeated assisted reproductive technology treatments and an unsuccessful outcome risk factors for unipolar depression in infertile women?. Acta Obstet. Gynecol. Scand..

[12] Vikström J., Josefsson A., Bladh M., Sydsjö G. (2015). Mental health in women 20–23 years after IVF treatment: A Swedish cross-sectional study. BMJ Open.

[13] Kim S.Y. (2016). Emotional labor of Chosun Dinasty women and their sharing of self-essay by mirroring. Korean Assoc. Fem. Philos..

[14] Kim U.J. (2015). The Effect of Self Disclosure on Posttraumatic Growth in Glaucoma Patients: Mediating Role of Social Support and Intentional Rumination. Korean J. Gestalt Counseli..

[15] Slade P., O’Neill C., Simpson A.J., Lashen H. (2007). The relationship between perceived stigma, disclosure patterns, support and distress in new attendees at an infertility clinic. Hum. Reprod..

[16] Cho M.J., Kim K.H. (1993). Diagnostic validity of the CES-D (Korean version) in the assessment of DSM-III-R major depression. J. Korean Neuropsychiat. Assoc..

[17] Boivin J., Takefman J., Braverman A.M. (2011). The fertility quality of life (FertiQoL) tool: Development and general psychometric properties. Hum. Reprod..

[18] Kahn J.H., Hessling R.M. (2001). Measuring the Tendency to Conceal Versus Disclose Psychological Distress. J. Soc. Clin. Psychol..

[19] Song H., Lee Y.S. (2013). The Mediating Effects of Emotional Self-Disclosure on the Relationship between Avoidance Attachment and Posttraumatic Growth. Korean J. Counseli..

[20] Baron R.M., Kenny D.A. (1986). The moderator–mediator variable distinction in social psychological research: Conceptual, strategic, and statistical considerations. J. Pers. Soc. Psychol..

[21] Taebi M., Kariman N., Montazeri A., Majd H.A. (2020). Development and psychometric evaluation of the female infertility stigma instrument (ISI-F): Protocol for a mixed method study. Reprod. Health.

[22] Jing X., Gu W., Xu X., Yan C., Jiao P., Zhang L., Li X., Wang X., Wang W. (2020). Stigma predicting fertility quality of life among Chinese infertile women undergoing in vitro fertilization–embryo transfer. J. Psychosom. Obstet. Gynecol..

[23] Kim M., Hong J.E., Lee E.Y. (2019). The Relationship between Fatigue, Health-Promoting Behavior, and Depression among Infertile Women. Korean J. Women Health Nurs..

[24] Hwang N., Jang I. (2015). Factors Influencing the Depression Level of Couples Participating in the National Supporting Program for Infertile Couples. J. Korean Acad. Community Health Nurs..

[25] Kim M., Kim H.S. (2018). Mediator effect of marital intimacy on the relationship between depression and marital satisfaction of infertile women. J. Korean Public Health Nurs..

[26] Kim M.O., Park J.S., Nam H.A. (2016). Factors Associated with Marital Satisfaction of Women under Infertility Treatments. J. Korean Soc. Matern. Child Health.

[27] Maroufizadeh S., Ghaheri A., Samani R.O. (2016). Factors associated with poor quality of life among Iranian infertile women undergoing IVF. Psychol. Health Med..

[28] Kim M., Nam H., Youn M. (2016). Infertility Stress, Depression, and Resilience in Women with Infertility treatments. J. Korean Public Health Nurs..

[29] Kim K.H. (2019). Effects on Posttraumatic Growth on Women Family Caregivers of Adults with Cancer Patients. Ph.D. Thesis.

[30] Yang S.R., Yeo J.H. (2017). Effects of Irrational Parenthood Cognition, Post Traumatic Stress Disorder and Spousal Support on Quality of Life of Infertile Women. Korean J. Women Health Nurs..

[31] Karaca A., Yavuzcan A., Batmaz S., Cangür Ş., Çalişkan A. (2019). The Effect of Cognitive Behavioral Group Therapy on Infertility Stress, General Health, and Negative Cognitions: A Randomized Controlled Trial. J. Ration. Cogn. Ther..

[32] Montgomery N.D. (2021). Uncovering In-Vitro Fertilization (IVF) Patient and Peer Relationships: A Qualitative Study on Self-Disclosure Processes in a Social Support Setting. Ph.D. Thesis.

[33] Steuber K.R., High A. (2015). Disclosure strategies, social support, and quality of life in infertile women. Hum. Reprod..

